# Supplementary material for: Quality of life and cognitive assessment in healthy older Asian people with early and moderate chronic kidney disease: The NAHSIT 2013–2016 and validation study
Source: PLoS One. 2022 Mar 10;17(3):e0264915. doi: 10.1371/journal.pone.0264915 (PMC8912208; doi:10.1371/journal.pone.0264915)
Supplement: S1 Table — (DOCX) [file pone.0264915.s001.docx]

**S1 Table. Mini-Mental State Examination and Quality of Life Assessment Stratified by Sex.**

|  | Male (*N* =284) | | | | | | Female (*N* =213) | | | | | | | | | | |  |
| --- | --- | --- | --- | --- | --- | --- | --- | --- | --- | --- | --- | --- | --- | --- | --- | --- | --- | --- |
| CKD Staging | Stage 1  (*N* = 20) | Stage 2  (*N* = 173) | Stage 3a, 3b  (*N* = 86) | Stage 4-5  (*N* = 5) | *P* value | *P* trend | Stage 1  (*N* = 46) | | Stage 2  (*N* = 130) | | Stage 3a, 3b  (*N* = 33) | | Stage 4-5  (*N = 4*) | | *P* value | | *P* trend |  |
| Global MMSE (30 points) | 27.4 ± 2.0 | 27.8 ± 2.4 | 26.6 ± 2.8 | 24.6 ± 3.4 | 0.0004* | 0.0006* | 27.5 ± 2.7 | | 26.7 ± 3.1 | | 22.0 ± 6.6 | | 25.3 ± 3.9 | | <0.0001* | | <0.0001* |  |
| Orientation to time | 4.8 ± 0.4 | 4.8 ± 0.4 | 4.7 ± 0.6 | 4.2 ± 1.1 | 0.0167 | 0.0204* | 4.9 ± 0.4 | | 4.8 ± 0.5 | | 4.1 ± 1.4 | | 4.5 ± 0.6 | | <0.0001* | | <0.0001* |  |
| Orientation to place | 5.0 ± 0.2 | 5.0 ± 0.1 | 5.0 ± 0.2 | 5.0 ± 0.0 | 0.7326 | 0.8338 | 4.9 ± 0.4 | | 4.9 ± 0.4 | | 4.5 ± 1.2 | | 5.0 ± 0.0 | | 0.0024* | | 0.0154* |  |
| Registration | 3.0 ± 0.0 | 3.0 ± 0.2 | 3.0 ± 0.2 | 2.8 ± 0.4 | 0.2328 | 0.6267 | 2.9 ± 0.5 | | 3.0 ± 0.2 | | 2.7 ± 0.8 | | 2.8 ± 0.5 | | 0.0057* | | 0.0333* |  |
| Calculation | 3.9 ± 1.3 | 4.2 ± 1.3 | 3.7 ± 1.5 | 2.4 ± 1.5 | 0.0017* | 0.0038* | 4.2 ± 1.3 | | 3.8 ± 1.5 | | 2.3 ± 1.6 | | 3.5 ± 1.9 | | <0.0001* | | <0.0001* |  |
| Memory recall | 2.5 ± 0.9 | 2.5 ± 0.7 | 2.3 ± 0.8 | 2.0 ± 1.0 | 0.1428 | 0.0585 | 2.5 ± 0.8 | | 2.3 ± 0.8 | | 1.5 ± 1.3 | | 2.5 ± 1.0 | | <0.0001* | | 0.0004 |  |
| Language | 2.0 ± 0.0 | 2.0 ± 0.2 | 2.0 ± 0.2 | 2.0 ± 0.0 | 0.9255 | 0.5902 | 2.0 ± 0.1 | | 2.0 ± 0.1 | | 1.9 ± 0.5 | | 2.0 ± 0.0 | | 0.1085 | | 0.1499 |  |
| Repetition | 0.9 ± 0.3 | 0.9 ± 0.3 | 0.8 ± 0.4 | 1.0 ± 0.0 | 0.2738 | 0.2377 | 0.9 ± 0.3 | | 0.9 ± 0.3 | | 0.9 ± 0.3 | | 0.5 ± 0.6 | | 0.1222 | | 0.2547 |  |
| Complex commands | 5.4 ± 0.7 | 5.4 ± 0.8 | 5.2 ± 1.1 | 5.2 ± 0.8 | 0.1938 | 0.0869 | 5.2 ± 1.1 | | 5.0 ± 1.3 | | 4.1 ± 1.6 | | 4.5 ± 1.0 | | 0.0006* | | 0.0004* |  |
| Quality of life assessment | |  |  |  |  |  |  |  | |  | |  | |  | |  | | |
| Role-physical (RP) | 54.7 ± 7.9 | 51.5 ± 9.0 | 48.6 ± 10.4 | 45.1 ± 12.8 | 0.0150* | 0.0012* | 49.7 ± 10.3 | | 49.3 ± 10.3 | | 48.0 ± 11.2 | | 40.3 ± 12.1 | | 0.3472 | | 0.1882 |  |
| Vitality (VT) | 54.3 ± 10.0 | 50.8 ± 9.5 | 50.3 ± 9.8 | 51.3 ± 11.4 | 0.4111 | 0.2423 | 50.3 ± 9.6 | | 49.2 ± 9.6 | | 45.6 ± 13.5 | | 39.4 ± 7.0 | | 0.0654 | | 0.0159* |  |
| Social functioning (SF) | 48.4 ± 12.0 | 50.5 ± 9.0 | 48.4 ± 11.6 | 50.9 ± 7.4 | 0.4185 | 0.4704 | 49.6 ± 11.3 | | 51.3 ± 8.3 | | 49.0 ± 10.3 | | 35.7 ± 27.2 | | 0.0174* | | 0.2923 |  |
| Role-emotional (RE) | 53.1 ± 6.5 | 50.9 ± 9.1 | 49.9 ± 9.6 | 49.5 ± 13.7 | 0.5370 | 0.1654 | 47.6 ± 12.2 | | 49.6 ± 10.4 | | 48.5 ± 12.0 | | 48.0 ± 9.8 | | 0.7681 | | 0.7151 |  |
| Mental health (MH) | 52.8 ± 7.7 | 50.5± 10.3 | 50.1 ± 9.4 | 55.5 ± 2.9 | 0.4864 | 0.7004 | 49.1 ± 9.9 | | 49.6 ± 10.4 | | 48.1 ± 10.4 | | 43.2 ± 13.1 | | 0.5909 | | 0.4228 |  |

CKD, chronic kidney disease, MMSE, Mini-Mental State Examination; N, number. *Variables with statistical significance (*P* < 0.05)
