# Supplementary material for: Quality of life and cognitive assessment in healthy older Asian people with early and moderate chronic kidney disease: The NAHSIT 2013–2016 and validation study
Source: PLoS One. 2022 Mar 10;17(3):e0264915. doi: 10.1371/journal.pone.0264915 (PMC8912208; doi:10.1371/journal.pone.0264915)
Supplement: S2 Table — (DOCX) [file pone.0264915.s002.docx]

**S2 Table. Spearman Correlation Coefficients among All Items of the Mini-Mental State Examination and SF-12**

|  | OTT | OTP | Reg | Cal | MR | Lan | Rep | CC | RP | VT | SF | RE | MH |
| --- | --- | --- | --- | --- | --- | --- | --- | --- | --- | --- | --- | --- | --- |
| OTT | 1.000 | 0.123** | 0.090* | 0.192*** | 0.132** | 0.152*** | -0.038 | 0.226*** | 0.114* | 0.074 | 0.030 | 0.004 | 0.032 |
| OTP | 0.123** | 1.000 | 0.130** | 0.265*** | 0.198*** | 0.212*** | 0.079 | 0.280*** | 0.058 | 0.136** | 0.049 | 0.051 | 0.082 |
| Reg | 0.090* | 0.130** | 1.000 | 0.218** | 0.077 | 0.225*** | 0.180*** | 0.199*** | 0.105* | 0.109* | 0.047 | 0.084 | 0.062 |
| Cal | 0.192*** | 0.265*** | 0.218*** | 1.000 | 0.211*** | 0.106* | 0.147* | 0.374*** | 0.151*** | 0.113* | 0.064 | 0.097* | 0.086 |
| MR | 0.132** | 0.198*** | 0.077 | 0.211*** | 1.000 | 0.018 | 0.049 | 0.224*** | 0.039 | 0.088 | 0.046 | 0.075 | 0.076 |
| Lan | 0.152*** | 0.212*** | 0.225*** | 0.106* | 0.018 | 1.000 | 0.111* | 0.141** | -0.024 | -0.064 | -0.046 | -0.016 | -0.033 |
| Rep | -0.038 | 0.079 | 0.180*** | 0.147* | 0.049 | 0.111* | 1.000 | 0.062 | 0.075 | 0.184*** | 0.122** | 0.067 | 0.089 |
| CC | 0.226*** | 0.280*** | 0.199*** | 0.374*** | 0.0224*** | 0.141** | 0.062 | 1.000 | 0.174*** | 0.100* | 0.019 | 0.069 | 0.080 |
| RP | 0.114* | 0.058 | 0.105* | 0.151*** | 0.039 | -0.024 | 0.075 | 0.174*** | 1.000 | 0.446*** | 0.322*** | 0.488*** | 0.297*** |
| VT | 0.074 | 0.136** | 0.109* | 0.113* | 0.088 | -0.064 | 0.184*** | 0.100* | 0.446*** | 1.000 | 0.331*** | 0.327*** | 0.566*** |
| SF | 0.030 | 0.049 | 0.047 | 0.064 | 0.046 | -0.046 | 0.122** | 0.019 | 0.322*** | 0.331*** | 1.000 | 0.265*** | 0.323*** |
| RE | 0.004 | 0.051 | 0.084 | 0.097* | 0.075 | -0.016 | 0.067 | 0.069 | 0.488*** | 0.327*** | 0.265*** | 1.000 | 0.353*** |
| MH | 0.032 | 0.082 | 0.032 | 0.086 | 0.076 | -0.033 | 0.089 | 0.080 | 0.297*** | 0.566*** | 0.323*** | 0.353*** | 1.000 |

Cal, Calculation; CC, Complex commands; Lan, language; MH, Mental-health; MR, memory recall; OTP, Orientation to place; OTT, Orientation to time; RE, Role-emotional; Reg, Registration; Rep, repetition; RP, Role-physical; SF, Social functioning; VT, Vitality;

Statistical significance of *p* value < 0.05*, <0.01**, and <0.001***.
